# Exploring the relationship between movement and breathing regulation in Tai Chi practice among middle-aged and older men using a three-dimensional respiratory–movement model

**DOI:** 10.3389/fspor.2025.1657944

**Published:** 2025-12-08

**Authors:** Jiaqi Zheng, Yatai Chai, Xiaofei Huang, Meijin Hou, Xiangbin Wang

**Affiliations:** 1 College of Rehabilitation Medicine, Fujian University of Traditional Chinese Medicine, Fuzhou, China; 2 Key Laboratory of Orthopedics & Traumatology of Traditional Chinese Medicine and Rehabilitation Ministry of Education, Fujian University of Traditional Chinese Medicine, Fuzhou, China; 3 National-Local Joint Engineering Research Center of Rehabilitation Medicine Technology, Fuzhou, China

**Keywords:** Tai Chi, long-term practitioners, 3D motion analysis, breathing regulation, postural control

## Abstract

**Purpose:**

Postural control and respiration are closely integrated processes vital for stability in older adults. Although Tai Chi emphasizes coordinated breath and movement, quantitative evidence comparing neuromuscular and biomechanical adaptations between long-term and beginner practitioners remains limited. This study systematically examined differences in breathing regulation and postural control during Tai Chi movements across the two groups.

**Methods:**

A three-dimensional motion analysis system synchronized with force plates and surface electromyography was employed. Lower-limb kinematics, kinetics, muscle activity, and thoracoabdominal motion were assessed during four classical Tai Chi movements in 21 long-term and 21 beginner practitioners. Primary outcomes included respiratory parameters, joint range of motion (ROM), center of pressure (COP) displacement, and cross-correlation between respiratory volume and center of mass (COM) trajectory.

**Results:**

Compared with beginners, long-term practitioners exhibited slower, deeper breathing, greater lower-limb joint ROM, particularly in the sagittal and frontal planes, and larger anteroposterior COP displacement during single-leg stance (Kick with Heel). During Wild Horse's Mane, they showed significantly lower cross-correlation between breathing and COM displacement, indicating task-specific decoupling between respiration and posture.

**Conclusion:**

Long-term Tai Chi practice enhances motor control through optimized diaphragmatic breathing, improved joint flexibility, and adaptive coordination between respiration and posture. These findings provide systematic biomechanical and physiological evidence for the mechanisms by which Tai Chi may improve postural control and respiratory function.

## 1 Introduction

Postural control is a fundamental component of human movement, essential for daily activities and fall prevention in older adults. A reciprocal relationship exists between respiration and postural control. Changes in trunk volume during breathing influence the sway of the body's center of mass (COM) (1). When the COM shifts due to movement, the body must promptly adjust posture and activate core muscles to maintain stability (2). Consequently, the postural control system must counteract movement-induced perturbations and reduce the influence of breathing on COM sway. Notably, breath regulation is a core component of various mind-body practices (e.g., yoga and Pilates) and respiratory rehabilitation programs aimed at enhancing stability and motor performance (3).

Commonly advocated breathing techniques include thoracic and diaphragmatic breathing (4). Diaphragmatic breathing emphasizes diaphragmatic excursion, enhancing ventilation and concurrently increasing intra-abdominal pressure. This stiffens the trunk and reduces postural sway (5). By maintaining the COM within a smaller, safer oscillation range, this mechanism improves balance. Such balance improvements align with the objectives of training paradigms that enhance sensorimotor integration (6).

Tai Chi (TC), a traditional Chinese mind-body exercise, integrates these elements by synchronizing breathing with multi-directional movements. Its philosophy of “sinking Qi to the Dantian” promotes deep, slow, diaphragmatic breathing, which may enhance pulmonary ventilation and core stability (7). Long-term practitioners (LPs) of TC demonstrate improvements in joint range of motion (ROM), muscle coordination, and center of pressure (COP) control, key targets for geriatric rehabilitation and fall prevention (8–10). However, there is limited quantitative evidence on whether LPs achieve superior postural control through refined breath-movement coordination compared to beginner practitioners (BPs). Elucidating this relationship is crucial to understand the mechanisms of TC and inform rehabilitation strategies.

This study employed a three-dimensional (3D) motion analysis system to compare lower-limb kinematics, kinetics, muscle activity, and thoracoabdominal motion in TC movements between LPs and BPs. We hypothesized that LPs would demonstrate deeper and slower breathing, superior postural control, and optimized breath-posture interaction, illustrating the benefits of long-term TC practice.

## 2 Materials and methods

### 2.1 Participants

Ethical approval for this study was granted by the Ethics Committee of the Rehabilitation Hospital Affiliated with Fujian University of Traditional Chinese Medicine (Approval No. 2023YJS-006-02). All experimental procedures were conducted according to the principles outlined in the Declaration of Helsinki. Participants were recruited from local TC classes and community centers in Fuzhou, China, via flyers and referrals from TC instructors. This strategy aimed to enroll individuals actively engaged in TC practice. Following a detailed explanation of the study procedures, all eligible individuals provided written informed consent.

An *a priori* power analysis was performed using G Power software. Based on a repeated-measures *t*-test (*α* = 0.05, power = 0.8, effect size f = 0.9), the analysis indicated a minimum required sample size of 42 participants. An effect size of 0.9 was adopted as it represents a large magnitude according to conventional benchmarks (11), consistent with the substantial differences in motor skills expected between LPs and BPs.

The inclusion criteria were as follows: for LPs, a minimum of 10 years of TC training experience; for BPs, a basic proficiency in the simplified 24-form TC, assessed by a certified TC instructor. All participants were aged between 50 and 80 years and practiced the same TC style (Yang style). Exclusion criteria included any history of cardiovascular or cerebrovascular disease, neurological or musculoskeletal disorders, regular participation in other exercise programs, or lower-limb joint pain during or after TC practice. The study was restricted to male participants due to methodological requirements for marker placement, as described in the Procedures section. Participants were allocated to either the LP group or the BP group (*n* = 21 per group) based on the predefined criteria. The mean duration of TC practice was 16 years for LPs and 3 years for BPs.

### 2.2 Protocol design

**Figure 1:**
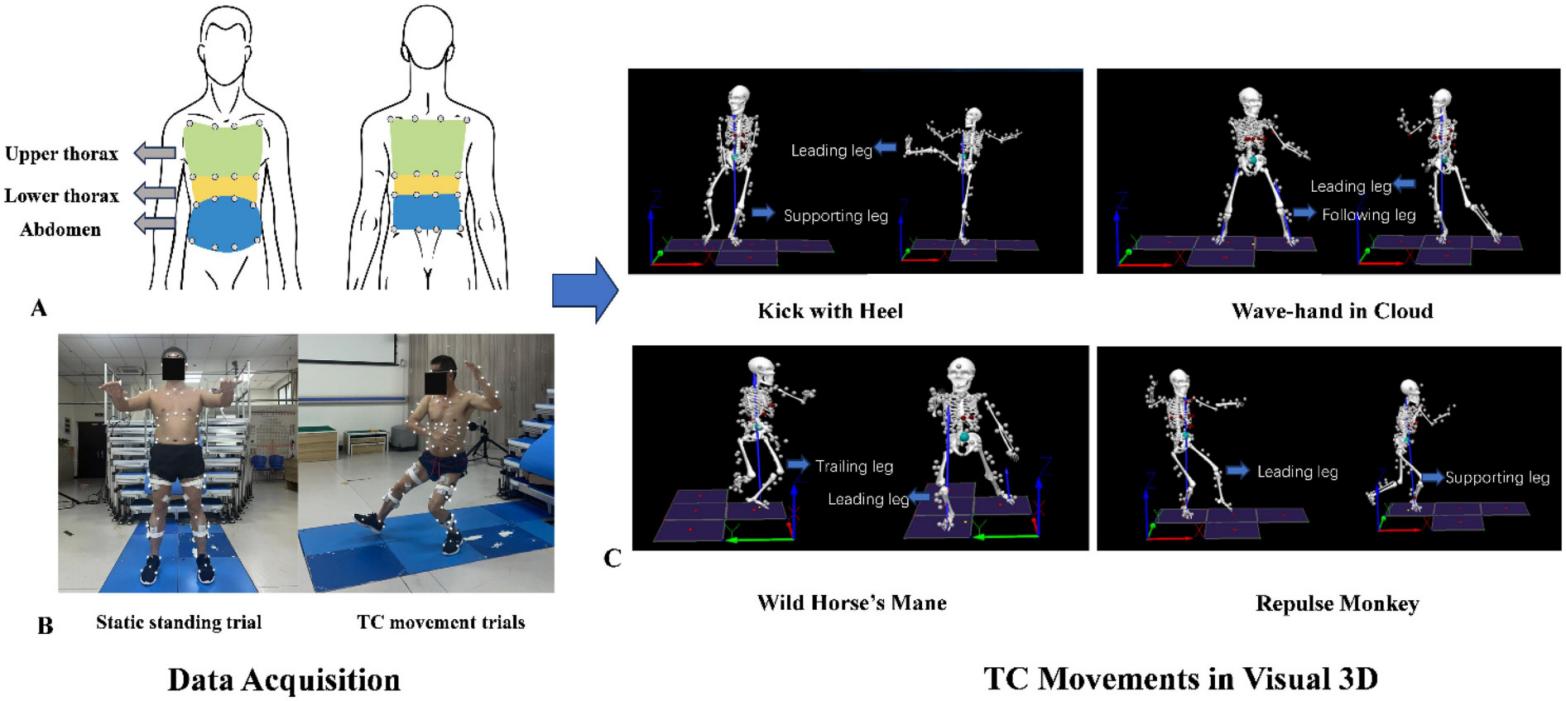
Experimental acquisition and analysis process in visual 3D. **(A)** 32-marker chest wall model and compartmentalization. **(B)** Experimental testing protocol. **(C)** Visual schematics of the four classical Tai Chi movements.

Movement kinematics were recorded at 100 Hz using a 12-camera motion capture system (Qualisys, Sweden) synchronized with four force plates (Kistler, Switzerland; model 9260AA; 60 cm × 50 cm) that captured COP data at 1,000 Hz. A 16-channel wireless surface electromyography (EMG) system (Delsys, USA; Trigno Avanti Sensor; 27 mm × 37 mm × 13 mm) simultaneously collected lower-limb muscle activity signals. Four classical TC movements were analyzed: “Kick with Heel” (KWH), “Wave-hand in Cloud” (WHIC), “Wild Horse's Mane” (WHM), and “Repulse Monkey” (RM). These movements encompass lateral, forward, and backward stepping as well as single-leg support, allowing a systematic assessment of multi-directional postural control and state-dependent respiratory coordination. Visual schematics and dynamic demonstrations of these movements are provided in Figure 1C and Supplementary Video S1, respectively (DOI: 10.6084/m9.figshare.30417175).

### 2.3 Procedures

To ensure reproducibility, standardized testing conditions were maintained. All participants were tested at the same time of day (±1 h) to control for diurnal variation and were instructed to avoid strenuous exercise for 24 h before testing. The experiments were conducted in a dedicated laboratory with controlled temperature, humidity, and lighting, a flat secure floor, and minimal vibration or auditory distraction. All data collection equipment was calibrated before testing.

Participants wore standardized athletic shorts and study-specific shoes. A total of 103 reflective markers (14 mm diameter) were attached according to the Calibrated Anatomical System Technique (CAST) (12) to define anatomical landmarks. This was complemented by a 32-marker 3D chest wall model (13), partitioned into upper thoracic, lower thoracic, and abdominal compartments for respiratory kinematics (14) (Figure 1A). Because the markers required direct skin placement on the chest and abdomen, and no prior data confirmed accurate tracking through clothing, the study was limited to male participants to avoid potential gender-related discomfort. Surface EMG sensors were attached to the following bilateral lower-limb muscles: gluteus maximus (GMAX), gluteus medius (GMED), rectus femoris (RF), vastus medialis (VM), vastus lateralis (VL), biceps femoris (BF), medial gastrocnemius (MG), and tibialis anterior (TA), following the SENIAM (Surface ElectroMyoGraphy for the Non-Invasive Assessment of Muscles) guidelines.

Before data collection, participants completed a standardized preparatory protocol that included light stretching and 2–3 familiarization sessions with the TC movements in the laboratory. This ensured adequate preparation and consistent performance across participants. The formal testing included a static calibration trial followed by the TC movement trials (Figure 1B). The static trial was used to generate a participant-specific anatomical model. Participants performed all TC movements at a self-selected comfortable pace, with at least three valid trials per movement. To prevent order effects such as fatigue or learning bias, the movement sequence was randomized for each participant. A minimum rest period of two minutes was provided between trials to reduce fatigue.

### 2.4 Blinding procedure

To minimize unconscious bias during data processing and analysis, a blinding procedure was implemented. Personnel responsible for pre-processing kinematic, kinetic, and EMG data were blinded to participant group assignments (LP vs. BP). All data files were anonymized and assigned unique codes before processing. Blinding was maintained until all statistical analyses were completed.

### 2.5 Data processing

Data processing was performed using Visual 3D software (C-Motion, Inc., USA). Lower-limb biomechanical parameters were extracted to evaluate postural control capacity. Respiratory parameters were computed to characterize breathing patterns and evaluate respiratory function. Furthermore, coupling between respiratory and postural control systems was quantified using the cross-correlation coefficient (CCF) between respiratory signals and COM trajectories.

Specifically, lower-limb kinematic measures included the ROM of the hip, knee, and ankle joints in the sagittal (flexion/extension), frontal (abduction/adduction), and transverse (internal/external rotation) planes. The specific roles of each lower limb during movements are illustrated in Figure 1C. Thoracoabdominal kinematics from the 3D chest wall model provided respiratory parameters, including respiratory volume, respiratory timing, and percentage contributions of each chest wall compartment to total volume. Kinetic analyses focused on COP displacement in the anterior-posterior (X) and medio-lateral (Y) directions, computed from force plate data. The trajectory of the COM was calculated from the kinematic model. To quantify coordination between breathing and postural control, CCFs were computed between respiratory volume signals and COM displacement along the X, Y, and superior-inferior (Z) axes for each movement cycle. Higher CCF values indicate stronger synchronization between respiratory and postural control signals.

Muscle activation was assessed using integrated electromyography (iEMG). Raw EMG signals were full-wave rectified, and iEMG values were calculated by integrating rectified signals over the entire movement cycle for each valid trial. For between-participant comparisons, iEMG values for each muscle were normalized to the peak amplitude observed during a reference voluntary contraction.

### 2.6 Statistical analysis

Statistical analyses were performed using SPSS software (version 25.0; IBM Corp., USA). Continuous variables are presented as mean ± standard deviation (SD) if normally distributed and as median with interquartile range (IQR; 25th–75th percentile) if non-normally distributed. Normality was assessed using the Shapiro–Wilk test. For each TC movement, at least three valid trials were collected per participant. Mean values across these trials represented participant performance for each movement, reducing within-participant variability and providing a stable estimate.

Between-group comparisons were conducted for the following five categories of outcome measures for each Tai Chi movement:

1. Respiratory Parameters: Including inspiratory time, expiratory time, maximum respiratory volume, and compartmental contributions (upper thorax, lower thorax, abdomen). These continuous variables were compared using independent samples *t*-tests for normally distributed data and Mann–Whitney *U*-tests for non-normally distributed data.
2. Lower-limb Joint ROM: Including the ROM of the bilateral hip, knee, and ankle joints in the sagittal, frontal, and transverse planes. These continuous variables were compared using independent samples *t*-tests for normally distributed data and Mann–Whitney *U*-tests for non-normally distributed data.
3. Muscle Activation: Including the iEMG values for the bilateral GMAX, GMED, RF, VM, VL, BF, MG, and TA. These continuous variables were compared using independent samples *t*-tests for normally distributed data and Mann–Whitney *U*-tests for non-normally distributed data.
4. COP Displacement: Including the COP displacement in the X and Y directions. These continuous variables were compared using independent samples *t*-tests for normally distributed data and Mann–Whitney *U*-tests for non-normally distributed data.
5. Breathing-Posture Coordination: Including the CCF between the respiratory volume signal and the COM displacement along the X, Y, and Z axes. These continuous variables were compared using independent samples *t*-tests for normally distributed data and Mann–Whitney *U*-tests for non-normally distributed data.

To account for multiple comparisons, the False Discovery Rate (FDR) correction method was applied. Statistical significance was determined based on FDR-corrected q-values (q < 0.05). For all between-group comparisons, effect sizes were calculated and reported: Hedges' g for normally distributed data and the rank-biserial correlation (r) for non-normally distributed data. Effect sizes are presented with their 95% confidence intervals (CIs) to quantify the magnitude and precision of observed effects.

## 3 Results

### 3.1 Basic information

The demographic and anthropometric characteristics of participants are summarized in Table 1. No significant between-group differences were observed in age, height, weight, body mass index (BMI), or proportions of smokers and alcohol consumers (all *P* > 0.05). A supplementary correlation analysis was performed to assess potential confounding effects from variations in TC practice duration within the LP group. The results indicated no significant correlations between years of TC practice and any primary outcome measures (all *P* > 0.1). Specifically, negligible correlations were observed with maximum respiratory volume (*ρ* = −0.11, *P* = 0.63) and knee joint sagittal-plane ROM of the leading leg during the KWH movement (*ρ* = −0.28, *P* = 0.23). These findings suggest that once a threshold of expertise (≥10 years) is attained, additional increases in practice duration may not substantially influence biomechanical or respiratory patterns. Consequently, the LP group can be considered functionally homogeneous regarding parameters of interest.

**Table 1:** Demographic and anthropometric of the participants.

Variables	LP group (*n* = 21)	BP group (*n* = 21)	*P*
Age (years)	62.43 ± 7.10	66.86 ± 7.21	0.05
Height (cm)	167.93 ± 6.17	168.52 ± 6.47	0.76
Weight (kg)	67.25 ± 9.43	70.67 ± 8.50	0.22
BMI (kg/m2)	23.81 ± 2.82	24.90 ± 2.82	0.22
Smokers [n(%)]	3 (14.29)	8 (38.10)	0.07
Alcohol consumers [n(%]	8 (38.10)	6 (28.57)	0.51

The data of age (years), height (cm), weight (kg) and BMI (kg/m^2^) were normal distribution and presented as mean ± SD. The data of smokers and alcohol consumers were non-normal distribution and presented as *n* (%). LP, long-term practitioners; BP, beginner practitioners.

Comprehensive data for all measured parameters across the four TC movements are available in the Supplementary Materials. A summary of statistically significant differences (q < 0.05) between LPs and BPs is presented in Table 2.

**Table 2:** Summary of statistically significant differences between LPs and BPs (q < 0.05).

Movement	Category	Parameters	LP Group (*n* = 21)	BP Group (*n* = 21)	Effect Size(95%CI)	q
KWH	Respiratory Time	Inspiratory Time (s)	1.28 ± 0.36	0.81 ± 0.37	1.26 (0.60,1.93)	0.002
	Respiratory Time	Expiratory Time (s)	2.04 ± 0.69	0.93 ± 0.58	1.71 (1.00,2.42)	<0.001
	COP Displacement (Supporting Leg)	X (m)	0.05 ± 0.01	0.04 ± 0.01	0.82 (0.19,1.45)	0.04
WHIC	Respiratory Time	Inspiratory Time (s)	1.07 (0.82,1.29)	0.63 (0.54,0.86)	0.58 (0.34,0.75)	0.01
	Respiratory Time	Expiratory Time (s)	1.40 (1.17,1.62)	0.99 (0.63,1.09)	0.51 (0.24,0.70)	0.02
	Leading Leg ROM	Ankle - Sagittal Plane (°)	43.42 ± 13.42	30.25 ± 11.06	1.05 (0.40,1.70)	0.01
	Leading Leg ROM	Ankle - Frontal Plane (°)	31.75 ± 7.16	23.82 ± 4.69	1.29 (0.62,1.95)	0.002
	Leading Leg ROM	Knee - Frontal Plane (°)	13.99 (11.90,17.71)	9.93 (8.31,12.03)	0.56 (0.31,0.74)	0.01
	Following Leg ROM	Ankle - Sagittal Plane (°)	53.98 ± 11.68	43.06 ± 9.74	1.00 (0.35,1.64)	0.01
	Following Leg ROM	Knee - Sagittal Plane (°)	46.04 ± 10.76	35.01 ± 7.26	1.18 (0.52,1.84)	0.004
	Following Leg ROM	Knee - Frontal Plane (°)	19.24 ± 6.79	13.30 ± 5.30	0.96 (0.32,1.60)	0.01
WHM	Respiratory Time	Inspiratory Time (s)	1.11 ± 0.35	0.74 ± 0.31	1.10 (0.45,1.75)	0.01
	Respiratory Time	Expiratory Time (s)	1.53 ± 0.42	0.92 ± 0.38	1.49 (0.81,2.18)	<0.001
	Leading Leg ROM	Knee - Sagittal Plane (°)	85.52 ± 15.50	70.82 ± 14.71	0.95 (0.31,1.59)	0.01
	Leading Leg ROM	Hip - Frontal Plane (°)	32.20 ± 9.66	24.04 ± 6.46	0.97 (0.33,1.62)	0.01
	Trailing Leg ROM	Ankle - Frontal Plane (°)	41.00 ± 5.41	31.16 ± 6.97	1.55 (0.85,2.24)	<0.001
	Trailing Leg ROM	Ankle - Transverse Plane (°)	20.05 ± 6.06	12.64 ± 4.59	1.35 (0.68,2.03)	0.001
	Trailing Leg ROM	Knee - Sagittal Plane (°)	56.89 ± 13.45	42.44 ± 13.62	1.05 (0.40,1.69)	0.01
	Trailing Leg ROM	Hip - Sagittal Plane (°)	58.53 ± 18.83	40.37 ± 19.27	0.94 (0.30,1.57)	0.02
	Trailing Leg ROM	Hip - Frontal Plane (°)	28.61 ± 6.72	20.37 ± 8.39	1.06 (0.42,1.71)	0.01
	CCF	X	0.46 (0.26,0.71)	0.76 (0.62,0.89)	0.48 (0.20,0.68)	0.04
	CCF	Z	0.54 ± 0.24	0.73 ± 0.16	−0.91(−1.55,−0.28)	0.02
RM	Respiratory Time	Inspiratory Time (s)	1.23 (1.05,1.36)	0.89 (0.77,1.07)	0.51 (0.24,0.70)	0.02
	Respiratory Time	Expiratory Time (s)	1.50 (1.24,1.79)	0.96 (0.80,1.15)	0.69 (0.49,0.82)	0.001
	Leading Leg ROM	Ankle - Sagittal Plane (°)	63.67 ± 15.29	50.47 ± 13.24	0.91(0.27,1.54)	0.04

Data are presented as mean ± SD for normally distributed variables and median (25th percentile, 75th percentile) for non-normally distributed variables. LP, long-term practitioners; BP, beginner practitioners. CCF, cross-correlation coefficient. Effect size (Hedges’g/r) and 95% confidence interval (CI) are reported. q-values represent false discovery rate (FDR)-corrected *p*-values. The X corresponds to the anterior-posterior direction, the Y to the medio-lateral direction, and the Z to the superior-inferior direction.

### 3.2 Respiratory parameters

As summarized in Table 2, LPs demonstrated significantly longer inspiratory and expiratory durations across all four movements compared to BPs (all q < 0.05). Although differences in maximum respiratory volume did not remain significant after FDR correction (all q > 0.05), a consistent trend indicated higher volumes among LPs across all movements (all uncorrected *P* < 0.05). Additionally, LPs showed a trend toward increased lower thoracic contributions to breathing (uncorrected *P* = 0.05 in KWH; uncorrected *P* = 0.04 in WHIC, WHM, and RM), with moderate effect sizes (Hedges' g > 0.6). These results suggest that long-term TC practice is associated with slower, deeper breathing and a shift toward diaphragmatic-dominant respiration.

### 3.3 Lower-limb joint ROM

LPs consistently demonstrated greater joint ROM during TC movements, particularly in the sagittal and frontal planes (Table 2). Following FDR correction, several significant differences emerged. During the WHIC movement, the leading leg exhibited significantly greater ROM at the ankle joint in both sagittal and frontal planes, and at the knee joint in the frontal plane. The following leg showed greater ROM at the knee joint in sagittal and frontal planes, and at the ankle joint in the sagittal plane. During the WHM movement, the leading leg demonstrated increased ROM at the knee joint in the sagittal plane and at the hip joint in the frontal plane. The trailing leg exhibited greater ROM at the ankle joint in frontal and transverse planes, at the knee joint in the sagittal plane, and at the hip joint in both sagittal and frontal planes. During the RM movement, the leading leg showed significantly increased ROM at the ankle joint in the sagittal plane.

### 3.4 Muscle activation

After applying FDR correction for multiple comparisons, no statistically significant between-group differences in muscle activation levels were detected (all q > 0.05). However, LPs exhibited increased muscle activation with moderate effect sizes (r = 0.30–0.40) in several movements, although these differences did not survive stringent correction criteria. For example, during the KWH movement, higher activation was observed in the TA, GMAX, and GMED of the leading leg and in the MG and BF of the support leg (uncorrected *P* < 0.05). Similar trends appeared in the VM of the leading leg during the WHIC movement, the GMAX of both legs and the VM of the trailing leg during the WHM movement, and the VM during the RM movement. These observations suggest latent divergences in muscular recruitment strategies; however, their reliability warrants further investigation in studies with larger sample sizes.

### 3.5 COP displacement

As presented in Table 2, during the KWH movement, the LP group exhibited significantly greater anteroposterior COP displacement in the supporting leg compared to the BP group (q = 0.04). No other significant between-group differences in COP displacement were observed in any other direction across the movements (all q > 0.05). Nominally significant differences (uncorrected *P* < 0.05) were also noted during the WHM and RM movements. Specifically, the LP group demonstrated greater anteroposterior COP displacement in the leading leg during the WHM movement and smaller anteroposterior COP displacement in the supporting leg during the RM movement.

### 3.6 Breathing-posture coordination

As detailed in Table 2, cross-correlation analyses revealed that during the WHM movement, the LP group exhibited significantly lower CCF values in the anterior-posterior (q = 0.04) and superior-inferior (q = 0.02) directions. A similar, non-significant trend was observed in the medio-lateral direction (q = 0.06). This indicates that during dynamic movements, LPs display reduced synchronization between breathing and postural sway. No significant between-group differences in CCF values were found in other movements after FDR correction.

## 4 Discussion

This study employed comprehensive 3D motion analysis to investigate differences in breathing patterns and postural control between LPs and BPs. The principal findings indicate that LPs, compared to BPs, exhibit distinct movement strategies characterized by deeper, slower, and more diaphragmatically dominant breathing, greater lower-limb joint ROM, larger COP displacements during stability-demanding single-leg stance tasks (indicative of a more active postural control strategy), and reduced synchronization between respiration and postural sway (COM displacement) during dynamic movements involving substantial weight shifts.

LPs demonstrated significantly longer respiratory durations and a tendency toward greater lower thoracic involvement, constituting a “deep-and-slow” breathing pattern consistent with the fundamental TC principle of “sinking Qi to the Dantian.” The 3D chest wall model identifies expansion of the lower thoracic compartment, housing the diaphragm and extending between the xiphoid process and the 10th rib, as an indicator of diaphragmatic breathing (15). A descent of the diaphragm by 1 cm contributes approximately 350 mL to ventilation (16). Thus, the diaphragm-driven, prolonged breathing strategy adopted by LPs likely facilitated increased respiratory volumes. This efficient diaphragmatic breathing pattern, sharing mechanistic similarities with stability principles in practices such as yoga and Pilates, may concurrently enhance dynamic postural control (17).

Furthermore, LPs exhibited significantly greater joint ROM, particularly in the sagittal and frontal planes, reflecting a movement strategy prioritizing flexibility and dynamic postural adjustments. This was especially evident during movements such as WHM and WHIC, which require substantial control of lateral and forward momentum. The increased joint ROM may enhance the capacity to absorb and manage postural perturbations, a key factor in fall prevention among older adults (18, 19).

A particularly noteworthy finding was that the significant decrease in breathing–posture synchronization (CCF) among LPs was observed only during the WHM movement, with no consistent pattern detected in other tasks. This finding suggests that the weakening of respiratory–postural coupling is task-dependent rather than universal, varying according to specific biomechanical demands. From a motor control perspective, WHM is a compound lunge movement requiring the integration of stability maintenance, momentum regulation, and directional change during substantial forward trunk displacement. This multi-component integration likely necessitates that the respiratory system minimize interference with postural control, or even strategically “yield priority”, to ensure fluidity and stability of motion. This interpretation aligns with Manor et al., who reported that stroke patients with impaired postural control exhibited stronger respiratory–postural synchronization, supporting the view that excessive inter-system coupling may indicate reduced postural stability (20). Therefore, the task-specific decoupling ability observed in this study may represent an adaptive neuromuscular advantage developed through long-term practice (21), suggesting that skilled practitioners can dynamically modulate coordination between physiological systems according to movement-specific demands.

Moreover, during the single-leg stance task of the KWH movement, LPs demonstrated greater anteroposterior COP displacement. This increased postural sway likely reflects not diminished stability but a more active exploratory strategy aimed at expanding stability boundaries and enhancing control redundancy (22, 23). Although most iEMG measures did not remain significant after FDR correction, observed trends of increased activation in muscles such as TA, GMAX, GMED, and BF, with moderate effect sizes, provide preliminary, hypothesis-generating support for this interpretation. These neuromuscular patterns suggest that long-term practice refines muscle recruitment, potentially enabling coordinated ankle–hip strategies that facilitate active postural control (24). Future studies with larger sample sizes are warranted to confirm these muscle activation patterns and clarify their functional role in balance regulation.

This study is among the first to employ 3D motion analysis for assessing respiratory parameters during TC. Several limitations should be acknowledged. First, including only male participants prevented examination of established gender differences in breathing patterns and postural control, limiting the generalizability of findings (25). Second, real-time neuromuscular regulation of respiration could not be fully elucidated due to the lack of direct diaphragmatic kinematic measurements. Third, the biomechanical analysis was restricted to four movements from the 24-form simplified TC routine. Although these movements systematically vary stepping directions and support patterns, they cannot fully capture breath–movement coordination throughout the entire sequence, potentially overlooking integrative aspects refined through long-term practice.

Addressing these limitations, future studies should prioritize enrolling female participants to achieve a comprehensive understanding of TC's effects. Additionally, integrating ultrasound imaging to directly track diaphragmatic motion could provide precise measurements of diaphragmatic engagement and its coordination with posture (26). Incorporating functional outcome measures, such as standardized balance assessments (e.g., Berg Balance Scale) and clinical respiratory tests (e.g., spirometry), would further clarify relationships between biomechanical parameters and tangible health benefits (27, 28). Expanding the sample size and investigating rehabilitation potential across diverse clinical populations (e.g., individuals with chronic obstructive pulmonary disease or histories of falls) will enhance understanding of respiratory and postural regulation in rehabilitation contexts.

## 5 Conclusions

In conclusion, this study provides systematic biomechanical and physiological evidence that long-term TC practice refines motor control. The identified characteristics, including deep, slow breathing, increased joint flexibility, greater COP displacement, and altered respiratory–postural coupling, collectively indicate a more efficient and adaptive motor control strategy in LPs. These findings offer mechanistic support for TC's use in fall prevention and pulmonary rehabilitation and highlight specific biomechanical targets for designing future individualized rehabilitation programs.

## Data Availability

The raw data supporting the conclusions of this article will be made available by the authors, without undue reservation.
