# Supplementary material for: Exploring the relationship between movement and breathing regulation in Tai Chi practice among middle-aged and older men using a three-dimensional respiratory–movement model
Source: Front Sports Act Living. 2025 Dec 8;7:1657944. doi: 10.3389/fspor.2025.1657944 (PMC12722886; doi:10.3389/fspor.2025.1657944)
Supplement: Supplementary file 1 [file Datasheet1.pdf]

Supplementary_Table_S1. Comparison of the respiratory-related parameters during TC movements

Movement	Parameters		LP group(n=21)	BP group(n=21)	P	Effect Size	95%CI	q
KWH	Maximum respiratory volume		1.29(0.93,1.57)	1.11(0.87,1.23)	0.01	0.38	(0.09,0.62)	0.08
	Compartment	Abdomen	31.83±5.02	34.05±4.54	0.14	-0.46	(-1.07,0.16)	0.24
		Lower Thorax	16.93±3.68	14.88±2.68	0.05	0.62	(0.00,1.24)	0.10
		Upper Thorax	51.24±4.92	51.07±3.96	0.90	0.04	(-0.57,0.64)	0.96
	Time	Inspiratory time	1.28±0.36	0.81±0.37	<0.001	1.26	(0.60, 1.93)	**0.002**
		Expiratory time	2.04±0.69	0.93±0.58	<0.001	1.71	(1.00, 2.42)	**<0.001**
WHIC	Maximum respiratory volume		1.25(0.86,1.52)	1.05(0.75,1.20)	0.004	0.44	(0.16,0.66)	0.06
	Compartment	Abdomen	31.75(30.06,33.78)	34.42(30.71,37.15)	0.11	0.25	(-0.06,0.51)	0.25
		Lower Thorax	16.91±3.64	14.84±2.47	0.04	0.65	(0.03,1.27)	0.10
		Upper Thorax	51.52±4.70	51.43±3.49	0.94	0.02	(-0.58,0.63)	0.98
	Time	Inspiratory time	1.07(0.82,1.29)	0.63(0.54,0.86)	<0.001	0.58	(0.34,0.75)	**0.01**
		Expiratory time	1.40(1.17,1.62)	0.99(0.63,1.09)	0.001	0.51	(0.24,0.70)	**0.02**
WHM	Maximum respiratory volume		1.25(0.91,1.46)	1.06(0.82,1.22)	0.009	0.40	(0.11,0.63)	0.06
	Compartment	Abdomen	19.02(15.29,25.98)	17.39(13.30,32.67)	0.47	0.11	(-0.20,0.40)	0.64
		Lower Thorax	16.92±3.78	14.76±2.51	0.04	0.66	(0.04,1.28)	0.10
		Upper Thorax	51.49±4.76	51.51±3.57	0.99	0.00	(-0.61,0.60)	0.99
	Time	Inspiratory time	1.11±0.35	0.74±0.31	0.001	1.10	(0.45,1.75)	**0.01**
		Expiratory time	1.53±0.42	0.92±0.38	<0.001	1.49	(0.81,2.18)	**<0.001**
RM	Maximum respiratory volume		1.25(0.95,1.43)	1.05(0.87,1.20)	0.01	0.40	(0.11,0.63)	0.06
	Compartment	Abdomen	31.91±4.93	33.99±4.36	0.16	-0.44	(-1.05, 0.17)	0.26
		Lower Thorax	16.92±3.60	14.82±2.53	0.04	0.66	(0.04, 1.28)	0.10
		Upper Thorax	51.17±4.64	51.19±3.53	0.99	0.00	(-0.61, 0.60)	0.99
	Time	Inspiratory time	1.23(1.05,1.36)	0.89(0.77,1.07)	0.001	0.51	(0.24, 0.70)	**0.02**
		Expiratory time	1.50(1.24,1.79)	0.96(0.80,1.15)	<0.001	0.69	(0.49, 0.82)	**0.001**

Data are presented as mean ± standard deviation for normally distributed variables and median (25th percentile, 75th percentile) for non-normally distributed variables. LP, long-term practitioners; BP, beginner practitioners. Effect size (Hedges' g/r) and 95% confidence interval (CI) are reported. q-values represent false discovery rate (FDR)-corrected p-values. Significant results after FDR correction ($q < 0.05$) are highlighted in bold.

Supplementary_Table_S2. Comparison of lower limb ROM during KWH and WHIC movements

Movement	Analysis side	Joint	Plane	LP group(n=21)	BP group(n=21)	P	Effect Size	95%CI	q
KWH	Leading leg	Ankle	sagittal	50.61±15.29	45.01±15.56	0.25	0.36	(-0.25,0.97)	0.38
			frontal	17.47(13.47,23.37)	17.89(12.77,19.64)	0.48	0.11	(-0.20,0.40)	0.64
			transverse	21.13±6.15	20.06±4.74	0.53	0.19	(-0.42,0.80)	0.63
		Knee	sagittal	101.18(90.98,106.25)	88.36(73.59,97.86)	0.02	0.36	(0.06,0.60)	0.09
			frontal	13.77(11.22,18.50)	14.31(10.93,18.18)	0.56	0.09	(-0.22,0.38)	0.67
			transverse	27.20±9.34	23.03±6.42	0.10	0.51	(-0.10,1.13)	0.18
		Hip	sagittal	68.73±10.85	62.19±14.57	0.11	0.50	(-0.12,1.11)	0.19
			frontal	27.38±8.13	22.87±8.27	0.08	0.54	(-0.08,1.16)	0.15
			transverse	15.48(10.85,20.12)	12.36(10.23,21.01)	0.65	0.07	(-0.24,0.37)	0.73
	Supporting leg	Ankle	sagittal	12.79±4.83	11.45±6.06	0.44	0.24	(-0.37,0.85)	0.54
			frontal	9.83(6.67,11.9)	7.24(5.77,9.31)	0.11	0.25	(-0.06,0.51)	0.25
			transverse	14.09±4.09	10.95±4.36	0.02	0.73	(0.10,1.35)	0.06
		Knee	sagittal	27.24±12.05	25.53±13.07	0.66	0.13	(-0.47,0.74)	0.76
			frontal	7.30(5.67,13.97)	7.73(5.08,9.43)	0.56	0.09	(-0.22,0.38)	0.67
			transverse	10.28(7.95,13.79)	9.44(8.15,11.91)	0.30	0.16	(-0.15,0.44)	0.45
		Hip	sagittal	33.48±11.75	35.39±11.99	0.61	-0.16	(-0.76,0.45)	0.71
			frontal	22.71±6.77	18.62±5.97	0.045	0.63	(0.01,1.25)	0.10
			transverse	17.43±6.20	16.03±5.43	0.44	0.24	(-0.37,0.84)	0.54
WHIC	Leading leg	Ankle	sagittal	43.42±13.42	30.25±11.06	0.001	1.05	(0.40,1.70)	**0.01**
			frontal	31.75±7.16	23.82±4.69	<0.001	1.29	(0.62,1.95)	**0.002**
			transverse	18.16±5.46	16.60±5.47	0.36	0.28	(-0.33,0.89)	0.47
		Knee	sagittal	46.92(34.71,56.47)	39.13(32.96,44.16)	0.07	0.28	(-0.03,0.54)	0.20
			frontal	13.99(11.90,17.71)	9.93(8.31,12.03)	<0.001	0.56	(0.31,0.74)	**0.01**
			transverse	21.76±6.27	20.09±4.85	0.34	0.29	(-0.32,0.90)	0.47
		Hip	sagittal	28.24(20.62,40.91)	20.85(18.24,23.30)	0.01	0.40	(0.11,0.63)	0.06
			frontal	31.59(26.80,36.80)	23.89(21.68,28.31)	0.01	0.40	(0.11,0.63)	0.06
			transverse	23.66(17.34,28.57)	16.17(13.90,23.18)	0.02	0.36	(0.06,0.60)	0.09
	Following leg	Ankle	sagittal	53.98±11.68	43.06±9.74	0.002	1.00	(0.35,1.64)	**0.01**
			frontal	32.88±6.31	28.00±6.33	0.02	0.76	(0.13,1.38)	0.06
			transverse	16.95±4.31	15.52±5.59	0.36	0.28	(-0.33, 0.89)	0.47
		Knee	sagittal	46.04±10.76	35.01±7.26	<0.001	1.18	(0.52, 1.84)	**0.004**
			frontal	19.24±6.79	13.30±5.30	0.003	0.96	(0.32, 1.60)	**0.01**
			transverse	26.93±6.66	22.97±6.97	0.07	0.57	(-0.05, 1.19)	0.14
		Hip	sagittal	30.33(24.02,37.01)	22.71(17.53,30.18)	0.02	0.36	(0.06, 0.60)	0.09
			frontal	30.95±6.91	27.57±4.52	0.07	0.57	(-0.05, 1.19)	0.14
			transverse	20.35(15.55,25.17)	20.98(16.31,24.85)	0.82	0.04	(-0.27, 0.34)	0.87

Data are presented as mean ± standard deviation for normally distributed variables and median (25th percentile, 75th percentile) for non-normally distributed variables. LP, long-term practitioners; BP, beginner practitioners. Effect size (Hedges' g/r) and 95% confidence interval (CI) are reported. q-values represent false discovery rate (FDR)-corrected p-values. Significant results after FDR correction ($q < 0.05$) are highlighted in bold.

Supplementary_Table_S3. Comparison of lower limb ROM during WHM and RM movements

Movement	Analysis side	Joint	Plane	LP group(n=21)	BP group(n=21)	P	Effect Size	95%CI	q
WHM	Leading leg	Ankle	sagittal	42.26±11.42	35.05±12.73	0.06	0.58	(-0.03,1.20)	0.13
			frontal	19.32(15.67,24.85)	16.27(15.09,18.64)	0.03	0.34	(0.03,0.58)	0.11
			transverse	15.08(11.84,23.59)	12.59(10.78,17.32)	0.16	0.22	(-0.09,0.49)	0.33
		Knee	sagittal	85.52±15.50	70.82±14.71	0.003	0.95	(0.31,1.59)	**0.01**
			frontal	15.37(11.18,19.04)	12.28(8.67,15.76)	0.02	0.36	(0.06,0.60)	0.09
			transverse	25.50±6.74	23.14±4.62	0.19	0.40	(-0.21,1.01)	0.30
		Hip	sagittal	39.44(30.76,47.80)	29.95(26.99,35.43)	0.01	0.40	(0.11,0.63)	0.06
			frontal	32.20±9.66	24.04±6.46	0.003	0.97	(0.33,1.62)	**0.01**
			transverse	22.67±6.06	20.52±8.36	0.35	0.29	(-0.32,0.90)	0.47
	Trailing leg	Ankle	sagittal	19.43(13.07,25.18)	18.77(16.25,23.11)	0.76	0.05	(-0.26,0.35)	0.82
			frontal	41.00±5.41	31.16±6.97	<0.001	1.55	(0.85,2.24)	**<0.001**
			transverse	20.05±6.06	12.64±4.59	<0.001	1.35	(0.68,2.03)	**0.001**
		Knee	sagittal	56.89±13.45	42.44±13.62	0.001	1.05	(0.40,1.69)	**0.01**
			frontal	12.30(9.91,15.16)	9.45(7.04,17.56)	0.18	0.21	(-0.10,0.48)	0.35
			transverse	16.92±7.39	13.91±5.69	0.15	0.45	(-0.16,1.06)	0.25
		Hip	sagittal	58.53±18.83	40.37±19.27	0.004	0.94	(0.30,1.57)	**0.02**
			frontal	28.61±6.72	20.37±8.39	0.001	1.06	(0.42,1.71)	**0.01**
			transverse	20.56(17.31,28.16)	19.72(12.55,26.06)	0.50	0.10	(-0.21,0.40)	0.64
RM	Leading leg	Ankle	sagittal	63.67±15.29	50.47±13.24	0.01	0.91	(0.27,1.54)	**0.04**
			frontal	27.89(24.55,34.08)	23.58(19.79,28.79)	0.07	0.28	(-0.03,0.54)	0.20
			transverse	20.26±5.53	16.62±5.17	0.03	0.67	(0.05,1.29)	0.08
		Knee	sagittal	65.35±9.85	58.24±10.34	0.03	0.69	(0.07,1.31)	0.08
			frontal	15.87±5.25	15.90±6.19	0.99	-0.01	(-0.61,0.60)	0.99
			transverse	25.80±5.59	25.44±6.24	0.85	0.06	(-0.55,0.66)	0.92
		Hip	sagittal	46.25±8.00	41.20±5.91	0.03	0.70	(0.08,1.33)	0.08
			frontal	30.09(24.65,34.55)	26.06(22.09,32.87)	0.17	0.21	(-0.10, 0.48)	0.34
			transverse	29.65±7.52	26.09±6.04	0.10	0.51	(-0.10,1.13)	0.18
	Supporting leg	Ankle	sagittal	62.91(59.27,68.33)	53.87(50.75,61.45)	0.004	0.44	(0.16,0.66)	0.06
			frontal	27.48±9.08	25.16±7.22	0.37	0.28	(-0.33,0.89)	0.47
			transverse	24.00±5.03	22.28±5.37	0.29	0.32	(-0.28,0.93)	0.42
		Knee	sagittal	46.28±6.62	40.57±8.47	0.02	0.74	(0.11,1.36)	0.06
			frontal	18.06±7.39	14.64±4.78	0.08	0.54	(-0.08,1.16)	0.15
			transverse	22.20±6.67	21.61±5.71	0.76	0.09	(-0.51,0.70)	0.86
		Hip	sagittal	26.97(23.60,32.34)	27.07(25.20,30.22)	0.86	0.03	(-0.28,0.33)	0.89
			frontal	34.04±9.15	31.15±7.90	0.28	0.33	(-0.28,0.94)	0.41
			transverse	31.37±5.57	28.90±5.51	0.16	0.44	(-0.17, 1.05)	0.26

Data are presented as mean ± standard deviation for normally distributed variables and median (25th percentile, 75th percentile) for non-normally distributed variables. LP, long-term practitioners; BP, beginner practitioners. Effect size (Hedges' g/r) and 95% confidence interval (CI) are reported. q-values represent false discovery rate (FDR)-corrected p-values. Significant results after FDR correction ($q < 0.05$) are highlighted in bold.

Supplementary_Table_S4. Comparison of lower limb iEMG during KWH and WHIC movements

Movement	Analysis side	Lower limb muscle	LP group(n=21)	BP group(n=21)	P	Effect Size	95%CI	q
KWH	Leading leg	TA	0.75(0.58,1.16)	0.50(0.25,0.80)	0.03	0.34	(0.03,0.58)	0.11
		VL	0.72(0.39,1.06)	0.55(0.25,0.70)	0.23	0.19	(-0.13,0.46)	0.39
		RF	2.68(1.57,3.82)	2.05(0.95,3.15)	0.13	0.23	(-0.08,0.50)	0.28
		VM	0.36(0.12,0.53)	0.15(0.03,0.41)	0.09	0.26	(-0.05,0.52)	0.24
		MG	0.11(0.09,0.21)	0.15(0.08,0.27)	0.43	0.12	(-0.19,0.41)	0.60
		BF	0.23(0.15,0.36)	0.20(0.13,0.32)	0.68	0.06	(-0.25,0.36)	0.75
		GMAX	0.47(0.33,0.68)	0.25(0.17,0.60)	0.04	0.32	(0.01,0.57)	0.14
		GMED	1.26(0.71,1.99)	0.78(0.48,1.18)	0.03	0.34	(0.03,0.58)	0.11
	Supporting leg	TA	0.97(0.71,1.50)	0.67(0.49,1.46)	0.07	0.28	(-0.03,0.54)	0.20
		VL	1.17(0.81,1.95)	0.99(0.60,1.65)	0.36	0.14	(-0.17,0.43)	0.52
		RF	0.72(0.45,1.70)	0.73(0.38,1.01)	0.57	0.09	(-0.22,0.38)	0.67
		VM	1.06(0.51,1.43)	0.70(0.46,0.95)	0.07	0.28	(-0.03,0.54)	0.20
		MG	1.02(0.82,1.64)	0.58(0.30,1.02)	0.02	0.36	(0.06,0.60)	0.09
		BF	0.92(0.60,1.33)	0.53(0.26,0.83)	0.02	0.36	(0.06,0.60)	0.09
		GMAX	1.79(0.90,2.84)	1.15(0.75,1.73)	0.08	0.27	(-0.04,0.53)	0.21
		GMED	1.73(0.80,2.61)	1.39(0.99,1.77)	0.49	0.11	(-0.20,0.40)	0.64
WHIC	Leading leg	TA	0.39(0.20,0.58)	0.29(0.12,0.55)	0.30	0.16	(-0.15,0.44)	0.45
		VL	0.77(0.28,1.40)	0.60(0.23,0.79)	0.29	0.16	(-0.15,0.45)	0.45
		RF	0.37(0.28,0.73)	0.31(0.17,0.46)	0.11	0.25	(-0.06,0.51)	0.25
		VM	0.55(0.25,1.16)	0.22(0.07,0.45)	0.01	0.40	(0.11,0.63)	0.06
		MG	0.24(0.13,0.36)	0.18(0.10,0.29)	0.19	0.20	(-0.11,0.48)	0.35
		BF	0.16(0.09,0.29)	0.15(0.07,0.27)	0.59	0.08	(-0.23,0.38)	0.68
		GMAX	0.26(0.17,0.61)	0.26(0.10,0.32)	0.19	0.20	(-0.11,0.48)	0.35
		GMED	0.42(0.28,0.76)	0.44(0.25,0.53)	0.57	0.09	(-0.22,0.38)	0.67
	Following leg	TA	0.24(0.17,0.41)	0.26(0.19,0.40)	0.89	0.02	(-0.28,0.32)	0.90
		VL	0.54(0.24,0.85)	0.38(0.28,0.89)	0.52	0.10	(-0.21,0.39)	0.66
		RF	0.31(0.17,0.53)	0.25(0.16,0.61)	0.85	0.03	(-0.28,0.33)	0.89
		VM	0.46(0.24,0.89)	0.28(0.18,0.56)	0.11	0.25	(-0.06,0.51)	0.25
		MG	0.37(0.26,0.51)	0.26(0.22,0.35)	0.08	0.27	(-0.04,0.53)	0.21
		BF	0.16(0.11,0.29)	0.16(0.08,0.22)	0.29	0.16	(-0.15, 0.45)	0.45
		GMAX	0.22(0.13,0.44)	0.17(0.12,0.22)	0.12	0.24	(-0.07,0.51)	0.27
		GMED	0.34(0.16,0.47)	0.33(0.28,0.52)	0.59	0.08	(-0.23, 0.38)	0.68

Data are presented as mean ± standard deviation for normally distributed variables and median (25th percentile, 75th percentile) for non-normally distributed variables. LP, long-term practitioners; BP, beginner practitioners. TA, tibialis anterior; VL, vastus lateralis; RF, rectus femoris; VM, vastus medialis; MG, medial gastrocnemius; BF, biceps femoris; GMAX, gluteus maximus; GMED, gluteus medius. Effect size (Hedges' g/r) and 95% confidence interval (CI) are reported. q-values represent false discovery rate (FDR)-corrected p-values. Significant results after FDR correction ($q < 0.05$) are highlighted in bold.

Supplementary_Table_S5. Comparison of lower limb iEMG during WHM and RM movements

Movement	Analysis side	Lower limb muscle	LP group(n=21)	BP group(n=21)	P	Effect Size	95%CI	q
WHM	Leading leg	TA	0.38(0.28,0.49)	0.37(0.21,0.51)	0.82	0.04	(-0.27,0.34)	0.87
		VL	0.58(0.27,0.97)	0.35(0.16,0.60)	0.57	0.09	(-0.22,0.38)	0.67
		RF	0.44(0.30,0.76)	0.42(0.28,0.77)	0.11	0.25	(-0.06,0.51)	0.25
		VM	0.42(0.23,0.97)	0.22(0.04,0.55)	0.04	0.32	(0.01,0.57)	0.14
		MG	0.15(0.06,0.23)	0.12(0.07,0.26)	0.87	0.03	(-0.28,0.33)	0.89
		BF	0.20(0.12,0.44)	0.21(0.11,0.31)	0.73	0.05	(-0.26,0.35)	0.79
		GMAX	0.51(0.29,0.74)	0.27(0.14,0.43)	0.03	0.34	(0.03,0.58)	0.11
		GMED	0.54(0.35,0.87)	0.34(0.25,0.75)	0.17	0.21	(-0.10,0.48)	0.34
	Trailing leg	TA	0.45(0.35,1.09)	0.47(0.20,0.96)	0.4	0.13	(-0.18,0.42)	0.57
		VL	1.07(0.79,1.64)	0.87(0.43,1.51)	0.23	0.19	(-0.13,0.46)	0.39
		RF	0.78(0.36,1.25)	0.54(0.29,1.00)	0.29	0.16	(-0.15,0.45)	0.45
		VM	1.02(0.72,1.70)	0.59(0.29,0.84)	0.01	0.40	(0.11,0.63)	0.06
		MG	0.18(0.11,0.42)	0.14(0.10,0.24)	0.35	0.14	(-0.17,0.43)	0.51
		BF	0.23(0.17,0.36)	0.15(0.11,0.25)	0.07	0.28	(-0.03,0.54)	0.20
		GMAX	0.36(0.19,0.70)	0.22(0.10,0.33)	0.01	0.40	(0.11,0.63)	0.06
		GMED	0.38(0.17,0.59)	0.33(0.26,0.59)	0.89	0.02	(-0.28,0.32)	0.90
RM	Leading leg	TA	0.58(0.43,1.02)	0.57(0.43,0.76)	0.47	0.11	(-0.20, 0.40)	0.64
		VL	0.73(0.38,1.17)	0.67(0.53,1.10)	0.95	0.01	(-0.30,0.31)	0.95
		RF	0.59(0.32,0.65)	0.38(0.30,0.60)	0.29	0.16	(-0.15,0.45)	0.45
		VM	0.54(0.26,0.99)	0.41(0.06,0.49)	0.02	0.36	(0.06,0.60)	0.09
		MG	0.85(0.66,1.29)	0.82(0.55,1.08)	0.15	0.22	(-0.09,0.49)	0.31
		BF	0.67(0.40,0.88)	0.71(0.48,0.86)	0.73	0.05	(-0.26,0.35)	0.79
		GMAX	0.57(0.35,0.70)	0.38(0.26,0.61)	0.22	0.19	(-0.12,0.47)	0.38
		GMED	0.56(0.36,1.04)	0.51(0.41,0.72)	0.49	0.11	(-0.20,0.40)	0.64
	Supporting leg	TA	0.56(0.43,0.76)	0.64(0.33,0.84)	0.54	0.10	(-0.22,0.39)	0.67
		VL	1.08(0.56,1.97)	1.01(0.59,1.39)	0.62	0.08	(-0.23,0.37)	0.71
		RF	0.94(0.38,1.29)	0.59(0.38,0.59)	0.49	0.11	(-0.20,0.40)	0.64
		VM	0.90(0.59,2.22)	0.54(0.43,1.08)	0.05	0.30	(0.00,0.56)	0.17
		MG	0.26(0.16,0.37)	0.30(0.21,0.39)	0.55	0.09	(-0.22,0.39)	0.67
		BF	0.31(0.25,0.59)	0.29(0.22,0.35)	0.31	0.16	(-0.16,0.44)	0.46
		GMAX	0.28(0.17,0.53)	0.18(0.15,0.36)	0.2	0.20	(-0.11,0.47)	0.36
		GMED	0.29(0.16,0.46)	0.39(0.28,0.69)	0.22	0.19	(-0.12,0.47)	0.38

Data are presented as mean ± standard deviation for normally distributed variables and median (25th percentile, 75th percentile) for non-normally distributed variables. LP, long-term practitioners; BP, beginner practitioners. TA, tibialis anterior; VL, vastus lateralis; RF, rectus femoris; VM, vastus medialis; MG, medial gastrocnemius; BF, biceps femoris; GMAX, gluteus maximus; GMED, gluteus medius. Effect size (Hedges' g/r) and 95% confidence interval (CI) are reported. q-values represent false discovery rate (FDR)-corrected p-values. Significant results after FDR correction ($q < 0.05$) are highlighted in bold.

Supplementary_Table_S6. Comparison of COP displacement during TC movements

Movement	Analysis side	Direction	LP group(n=21)	BP group(n=21)	P	Effect Size	95%CI	q
KWH	Supporting leg	X	0.05±0.01	0.04±0.01	0.01	0.82	(0.19,1.45)	**0.04**
		Y	0.06(0.05,0.07)	0.07(0.05,0.07)	0.50	0.10	(-0.21,0.40)	0.64
WHIC	Leading leg	X	0.06(0.04,0.07)	0.06(0.04,0.09)	0.27	0.17	(-0.14,0.45)	0.44
		Y	0.12±0.02	0.11±0.02	0.25	0.38	(-0.23,0.99)	0.38
	Following leg	X	0.03±0.02	0.03±0.01	0.48	0.06	(-0.54, 0.67)	0.58
		Y	0.13±0.02	0.12±0.02	0.82	0.22	(-0.38, 0.83)	0.91
WHM	Leading leg	X	0.19(0.18,0.21)	0.18(0.16,0.19)	0.01	0.40	(0.11,0.63)	0.06
		Y	0.06(0.05,0.07)	0.07(0.04,0.08)	0.63	0.07	(-0.24, 0.37)	0.71
	Trailing leg	X	0.06(0.05,0.09)	0.07(0.06,0.09)	0.35	0.14	(-0.17,0.43)	0.51
		Y	0.06(0.04,0.07)	0.05(0.03,0.06)	0.07	0.28	(-0.03,0.54)	0.20
RM	Supporting leg	X	0.08(0.06,0.11)	0.12(0.08,0.15)	0.02	0.36	(0.06, 0.60)	0.09
		Y	0.10(0.09,0.12)	0.09(0.08,0.11)	0.13	0.23	(-0.08, 0.50)	0.28

Data are presented as mean ± standard deviation for normally distributed variables and median (25th percentile, 75th percentile) for non-normally distributed variables. LP, long-term practitioners; BP, beginner practitioners. Effect size (Hedges' g/r) and 95% confidence interval (CI) are reported. q-values represent false discovery rate (FDR)-corrected p-values. Significant results after FDR correction ($q < 0.05$) are highlighted in bold. The X-axis corresponds to the anterior-posterior direction, the Y-axis to the medio-lateral direction.

Supplementary_Table_S7 Comparison of CCF during TC movements

Movement	CCF	LP group(n=21)	BP group(n=21)	P	Effect Size	95%CI	q
KWH	X	0.36±0.26	0.54±0.26	0.03	-0.68	(-1.30,-0.06)	0.08
	Y	-0.39(-0.55，-0.29)	-0.57(-0.76，-0.26)	0.10	0.25	(-0.05,0.52)	0.25
	Z	0.39(0.30, 0.55)	0.55(0.27, 0.77)	0.13	0.23	(-0.08,0.50)	0.28
WHIC	X	0.66(0.50,0.75)	0.71(0.62,0.84)	0.27	0.17	(-0.14, 0.45)	0.44
	Y	0.65(0.48,0.79)	0.76(0.63,0.85)	0.08	0.27	(-0.04, 0.53)	0.21
	Z	0.62±0.16	0.68±0.22	0.30	-0.31	(-0.91, 0.30)	0.42
WHM	X	0.46(0.26,0.71)	0.76(0.62,0.89)	<0.01	0.48	(0.20, 0.68)	**0.04**
	Y	0.43(0.30,0.72)	0.76(0.59,0.88)	0.01	0.40	(0.11, 0.63)	0.06
	Z	0.54±0.24	0.73±0.16	<0.01	-0.91	(-1.55, -0.28)	**0.02**
RM	X	0.73(0.41,0.84)	0.56(0.35,0.70)	0.20	0.20	(-0.11, 0.47)	0.36
	Y	0.53±0.29	0.52±0.23	0.85	0.04	(-0.57, 0.64)	0.92
	Z	0.68(0.51,0.82)	0.58(0.49,0.70)	0.19	0.20	(-0.11, 0.48)	0.35

Data are presented as mean ± standard deviation for normally distributed variables and median (25th percentile, 75th percentile) for non-normally distributed variables. LP, long-term practitioners; BP, beginner practitioners. CCF, cross-correlation coefficient. Effect size (Hedges' g/r) and 95% confidence interval (CI) are reported. q-values represent false discovery rate (FDR)-corrected p-values. Significant results after FDR correction ($q < 0.05$) are highlighted in bold. The X-axis corresponds to the anterior–posterior direction, the Y-axis to the medio-lateral direction, and the Z-axis to the superior–inferior direction.
